# Case report: Vogt-Koyanagi-Harada-like uveitis induced by anti-PD-1 therapy (Sintilimab) in a gastric cancer patient: a case study

**DOI:** 10.3389/fphar.2026.1857320

**Published:** 2026-07-06

**Authors:** Lei Wang, Ran Gao, Si-Yong Lin

**Affiliations:** Department of Ophthalmology, Beijing Tsinghua ChangGung Hospital, School of Clinical Medicine, Tsinghua Medicine, Tsinghua University, Beijing, China

**Keywords:** immune checkpoint inhibitor, PD-1, sintilimab, uveitis, Vogt-Koyanagi-Harada disease

## Abstract

**Background:**

Immune checkpoint inhibitors (ICIs), such as anti-programmed cell death-1 (PD-1) antibodies, have become pivotal in treating advanced cancers. While effective, they are associated with immune-related adverse events (irAEs). Ocular irAEs, including uveitis, are uncommon, and Vogt-Koyanagi-Harada (VKH)-like panuveitis is a rare but vision-threatening complication. Sintilimab is a PD-1 inhibitor used in China, and this report describes a case of VKH-like uveitis following its long-term use for gastric cancer.

**Case Presentation:**

A 55-year-old man presented with a 1-week history of bilateral blurred vision and tinnitus. He had been receiving sintilimab for metastatic gastric cancer over the past year. His best-corrected visual acuity (BCVA) was 0.3 (approximately 6/20) in the right eye and 0.15 (approximately 6/40) in the left. Ophthalmic examination revealed bilateral anterior uveitis, optic disc swelling, and peripheral choroidal detachments. Optical coherence tomography (OCT) showed retinal pigment epithelium (RPE) undulations, subretinal fluid, and choroidal thickening. Fundus Fluorescein Angiography (FFA) confirmed bilateral diffuse pinpoint leakage and late optic nerve hyper fluorescence. A diagnosis of VKH-like panuveitis was made. Sintilimab was discontinued, and treatment with topical prednisolone acetate was initiated. Within 4 weeks, the patient’s BCVA improved to 0.8 in both eyes, and this improvement remained stable at the 5-month follow-up.

**Conclusion:**

This case suggests that long-term therapy with sintilimab, a PD-1 antibody, can trigger VKH-like uveitis. Early recognition, prompt discontinuation of the ICI, and anti-inflammatory treatment are crucial for visual recovery. It highlights the necessity for ophthalmological monitoring in cancer patients on long-term immunotherapy, even after many months of treatment.

## Introduction

Programmed cell death-1 (PD-1) inhibitors, including sintilimab, have demonstrated significant efficacy in the treatment of various advanced malignancies, including gastric cancer. These agents work by blocking the PD-1 pathway, thereby enhancing T-cell-mediated immune responses against tumor cells (Xu et al., 2023). However, this enhanced immunity can also lead to a spectrum of immune-related adverse events (irAEs), affecting various organs (Puzanov et al., 2017). Ocular irAEs, though relatively rare, can include conditions such as uveitis, dry eye, and keratitis (Papavasileiou et al., 2016; Zhou and Wei, 2021). Vogt-Koyanagi-Harada (VKH) disease is a multisystem autoimmune disorder characterized by bilateral granulomatous panuveitis, often accompanied by exudative retinal detachments, tinnitus, and other extraocular manifestations. It is thought to result from an autoimmune response directed against melanocytes (Du et al., 2016; Sakata et al., 2014). There have been increasing reports of ICI-induced uveitis that closely mimics the clinical presentation of VKH disease (Kurono et al., 2020). The onset of such VKH-like uveitis can vary significantly, occurring anywhere from a few days to over 20 months after initiating ICI therapy (Zhou and Wei, 2021; Dermarkarian et al., 2020). Here, we present a case of VKH-like panuveitis that developed in a patient after 1 year of sintilimab treatment for metastatic gastric cancer. This case underscores the importance of vigilance for this potential complication, even after prolonged periods of treatment.

## Case presentation

A 55-year-old man was referred to the ophthalmology department with complaints of bilateral blurred vision and tinnitus persisting for 1 week. His medical history was significant for metastatic gastric adenocarcinoma, for which he had been receiving intravenous sintilimab (a PD-1 inhibitor) every 3 weeks for the past 12 months. He reported no prior ocular history or history of autoimmune disease.

**FIGURE 1:**
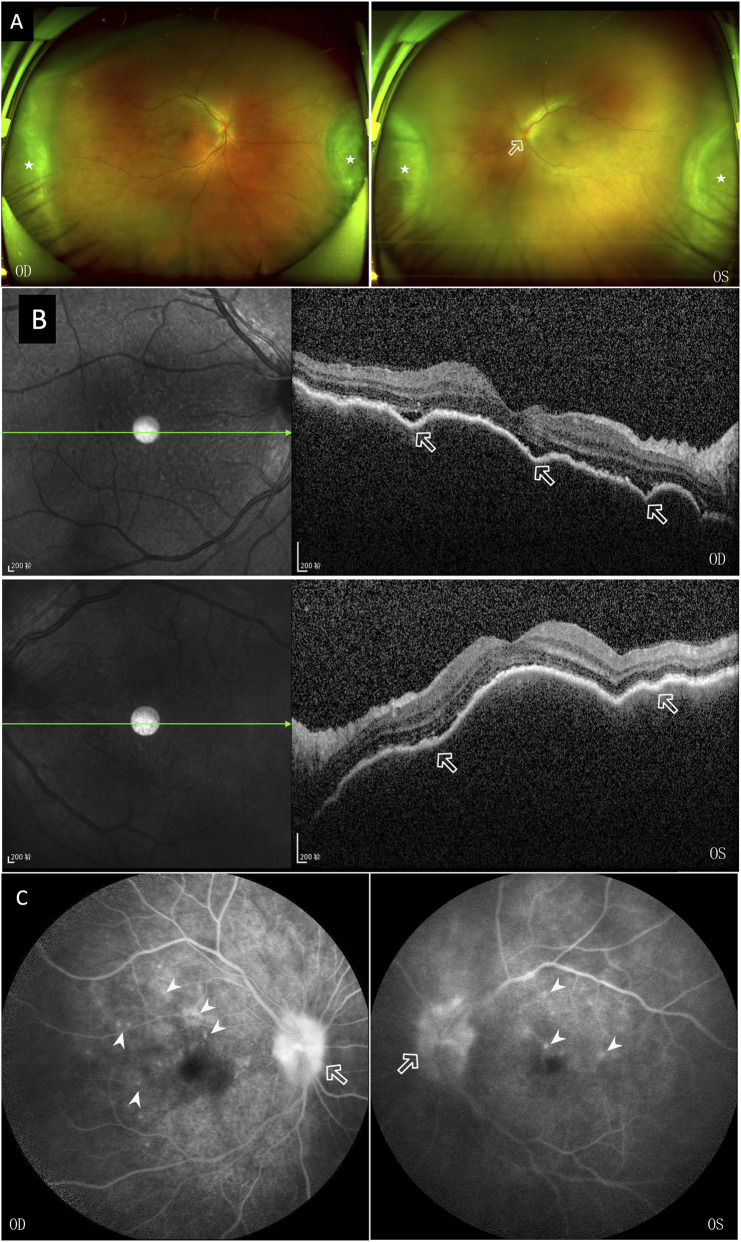
Panel **(A)**: Dilated fundus examination of both eyes revealed optic disc hyperemia and swelling (arrows) as well as peripheral serous choroidal detachments (asterisks). Panel **(B)**: OCT demonstrated wavy elevations of the RPE, subretinal fluid, and significant choroidal thickening in both eyes. Panel **(C)**: FFA showed early multiple pinpoint hyperfluorescent dots, which increased in intensity and coalesced in the later phases, creating a characteristic pattern of diffuse pinpoint leakage at the level of the RPE (arrowheads). Late-phase frames also revealed staining and leakage from the optic nerve heads (arrows).

Ophthalmic Examination: On examination, his best-corrected visual acuity (BCVA) was 0.3 (approximately 6/20) in the right eye and 0.15 (approximately 6/40) in the left eye. Intraocular pressures were within normal limits (11 mmHg in the right eye and 10 mmHg in the left). Slit-lamp examination revealed bilateral anterior chamber cells and flare (consistent with anterior uveitis). According to SUN grading criteria, anterior chamber cells were 1+ (6–15 cells per high-power field) in the right eye and 2+ (16–25 cells per high-power field) in the left eye. Dilated fundus examination of both eyes showed optic disc hyperemia and swelling (Figure 1A, arrow) and peripheral serous choroidal detachments (Figure 1A, asterisks). Vitreous haze was graded as 2+ in both eyes (SUN criteria: fundus details moderately blurred with optic nerve and vessels still visible). Optical coherence tomography (OCT) demonstrated wavy elevations of the retinal pigment epithelium (RPE), subretinal fluid, and significant choroidal thickening in both eyes (Figure 1B). FFA revealed early multiple pinpoint hyperfluorescent dots, which increased in intensity and coalesced in the later phases, creating a characteristic pattern of diffuse pinpoint leakage at the level of the RPE (Figure 1C, arrowheads). Late-phase frames also showed staining and leakage from the optic nerve heads (Figure 1C, arrows). These clinical and imaging findings are hallmark features of VKH syndrome.

Treatment and Clinical Course: A multidisciplinary decision was made in consultation with the oncology team to discontinue sintilimab permanently. The patient was started on intensive topical corticosteroid therapy (prednisolone acetate 1% eye drops, administered eight times daily) and a cycloplegic agent. Systemic corticosteroids were not administered due to the patient’s overall clinical status and the significant improvement observed with topical treatment alone. The patient’s symptoms and clinical signs improved rapidly. Within 4 weeks, the anterior chamber inflammation had resolved, the subretinal fluid on OCT had significantly decreased, and his BCVA improved to 0.8 (approximately 20/25) in both eyes. At the 5-month follow-up, his vision remained stable at 0.8 (approximately 20/25).

## Discussion

We report a case of VKH-like panuveitis that developed in a patient after 1 year of sintilimab therapy for gastric cancer. The diagnosis was based on characteristic clinical features, including bilateral panuveitis, exudative retinal detachments (evident as choroidal detachments and subretinal fluid on OCT), and typical angiographic findings on FFA. The temporal relationship between the drug administration and the onset of symptoms, along with the exclusion of other causes, strongly supports the diagnosis of an immune-related adverse event (Kumar et al., 2017).

Nevertheless, several alternative diagnoses warrant consideration. First, CSCR-like reactions lack significant intraocular inflammation and choroidal thickening, both present in our case. Second, posterior scleritis typically presents with ocular pain and scleral thickening on B-scan—both absent in our patient—and lacks the classic FFA findings of VKH disease. Third, paraneoplastic uveitis causes photoreceptor degeneration and retinal thinning without choroidal thickening or exudative detachments, which were key features of our case. Fourth, uveal effusion syndrome occurs without intraocular inflammation or typical FFA findings, and our patient had neither hyperopia nor scleral thickening. In summary, the combination of bilateral panuveitis, diffuse choroidal thickening, characteristic FFA findings, and treatment response supports ICI-induced VKH-like uveitis over these alternatives.

The pathogenesis of ICI-induced VKH-like uveitis is intricately linked to the disruption of ocular immune privilege, a state maintained in part by the PD-1/PD-L1 checkpoint pathway (Sugita et al., 2009). In the posterior segment, RPE cells and ocular melanocytes constitutively express PD-L1 (Charles et al., 2010). The interaction between PD-L1 and its receptor PD-1 on T cells delivers a potent inhibitory signal, crucial for suppressing intraocular immune responses and maintaining local tolerance (Ke et al., 2010; Sugita et al., 2013). By blocking this PD-1/PD-L1 interaction, sintilimab may unleash autoreactive T-cells. This is believed to trigger an autoimmune attack against melanocytes, targeting shared antigens present in the uvea, skin, and inner ear—a mechanism consistent with molecular mimicry, which underlies the phenotypic resemblance to classic VKH disease (Qian et al., 2024; Takeuchi et al., 2023; Sada et al., 2023). Our case underscores that this immune disruption can manifest after a prolonged period (12 months) of treatment, highlighting the necessity for sustained ophthalmic vigilance throughout PD-1 inhibitor therapy, even during extended treatment courses.

This case adds to the growing literature on this rare complication, which has been reported with other PD-1 inhibitors such as nivolumab (Obata et al., 2019) and the newer agent toripalimab (Wang et al., 2024). To our knowledge, this represents the first reported instance of Vogt-Koyanagi-Harada-like syndrome developing in a patient with gastric cancer following long-term (12 months) treatment with sintilimab. To date, no incidence rates for sintilimab-associated ocular complications have been established. However, at least one additional case of sintilimab-induced panuveitis has been reported in a pediatric patient with soft tissue sarcoma (Shen et al., 2023), suggesting that sintilimab can trigger various phenotypic expressions of intraocular inflammation. A notable feature of our case is the protracted treatment duration prior to symptom onset, underscoring that this adverse event can manifest after a substantial period of therapy and highlighting the necessity for sustained ophthalmic vigilance in patients receiving long-term ICI regimens.

Management of such cases involves a collaborative approach between oncologists and ophthalmologists. In our patient, systemic corticosteroids were not administered. According to the ASCO guideline (Schneider et al., 2021), for grade 1–2 uveitis, topical corticosteroids are recommended as first-line therapy. Our patient presented with non-severe disease and showed rapid clinical improvement within 72 h of initiating intensive topical therapy (prednisolone acetate 1% eight times daily). Given his advanced gastric cancer and ECOG performance status 2, we opted for topical monotherapy to avoid potential risks associated with systemic immunosuppression. According to guidelines, for severe ocular irAEs (Grade 3–4), permanent discontinuation of the ICI should be considered (Schneider et al., 2021). The mainstay of treatment is immunosuppression, typically with corticosteroids. Our patient showed an excellent response to topical corticosteroids alone after drug withdrawal. However, more severe cases may require systemic or intravitreal corticosteroids. The favorable visual outcome in this case highlights that early diagnosis and appropriate intervention can lead to significant visual recovery.

A limitation of this report is the lack of confirmatory follow-up OCT imaging, as the patient was followed remotely via telephone after transferring care to an outside hospital and later passed away.

## Conclusion

This case illustrates that sintilimab, like other PD-1 inhibitors, can induce a VKH-like panuveitis, even after long-term administration (1 year in this instance). It is crucial for both oncologists and ophthalmologists to be aware of this potential sight-threatening complication. Patients on PD-1 inhibitor therapy should be advised to report any visual changes or ocular symptoms immediately. Prompt ophthalmic evaluation, accurate diagnosis, discontinuation of the offending agent, and timely initiation of anti-inflammatory therapy are essential for preserving vision. This case underscores the importance of long-term ophthalmic monitoring in patients receiving immune checkpoint inhibition therapy.

## Data Availability

The original contributions presented in the study are included in the article/supplementary material, further inquiries can be directed to the corresponding authors.
